# Next Generation Pathology

**DOI:** 10.1007/s00292-024-01330-9

**Published:** 2024-04-25

**Authors:** Carolin Mogler

**Affiliations:** 1 Zentrum für Personalisierte Medizin (ZPM), Klinikum rechts der Isar, Technische Universität München, München, Deutschland; 2 Bayerisches Zentrum für Krebsforschung (BZKF), Standort Technische Universität München, München, Deutschland; 3 Deutsches Konsortium für Translationale Krebsforschung (DKTK), München, Deutschland; 4 Comprehensive Cancer Center München, Klinikum rechts der Isar, Technische Universität München, München, Deutschland; 5 Institut für Allgemeine Pathologie und Pathologische Anatomie, Technische Universität München, Trogerstraße 18, 81675 München, Deutschland

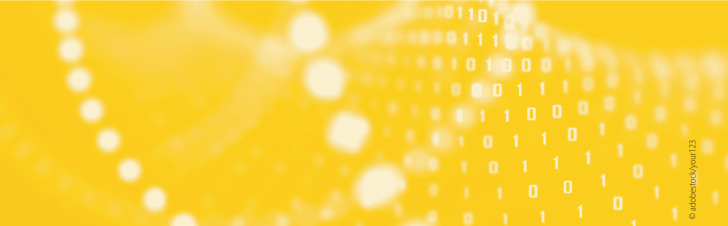



Liebe LeserInnen,

eigentlich sollte diese Einführung zum Sonderheft „Next Generation Pathology“ von Wilko Weichert verfasst sein. Wie Sie alle wissen, ist er leider noch deutlich vor Drucklegung dieser lange geplanten Ausgabe seiner schweren Erkrankung erlegen.

Daher ist es mir eine besondere Freude und große Ehre, diese Aufgabe für ihn zu übernehmen.

Wir haben insgesamt vier große Schwerpunkte für dieses Sonderheft als zukunftsträchtige Themen in der Pathologie definiert: Molekularpathologie, personalisierte Medizin, vergleichende Pathologie und digitale Pathologie.

Zu allen Themen wurden Beiträge überwiegend aus unserem Institut aber auch von externen Kollegen zusammengestellt. Von *Maria Walker und Nicole Pfarr* finden Sie u. a. einen spannenden Übersichtsartikel zum Thema „Molekularpathologie im Wandel der Zeit“. Zu diesem Thema gibt es auch einen (noch persönlich von Wilko Weichert eingeladenen) editorialen Gastbeitrag von *Prof. Manfred Dietel,* der sich ebenfalls mit der Entwicklung der Molekularpathologie in den letzten Jahrzehnten beschäftigt.

Für den Bereich der personalisierten Medizin konnten wir *Frau Dr. Alisa Lörsch* aus der Klinik und Poliklinik für Hämatologie und Onkologie, Klinikum rechts der Isar und *Prof. Lena Illert*, Leiterin des Zentrums für personalisierte Medizin (ZPM) am Klinkum rechts der Isar, beide München, gewinnen. Sie schildern uns die Entwicklung der ZPM aus internistisch-onkologischer Sicht und beleuchten aktuelle Herausforderungen und Chancen.

Der Bereich der vergleichenden Pathologie wurde vorrangig von unseren beiden Tierpathologinnen *Frau Dr. Tanja Groll und Frau PD Dr. Katja Steiger* adressiert mit dem Titel „Vergleichende Pathologie in der onkologischen Forschung“. In diesem Artikel finden Sie auch einige anschaulich dargestellte Bespiele zu Fallstricken in der experimentellen, tiermodellbasierten Forschung, die sicher für alle sehr lesenswert sind.

Zu guter Letzte haben sich *Prof. Peter Schüffler* und ich dem Thema der künstlichen Intelligenz in der Pathologie für den Bereich digitale Pathologie angenommen, eines der sicher sich am schnellsten weiter entwickelnden Felder im Bereich der „Next Generation Pathology“.

Ich wünsche Ihnen viel Spaß beim Lesen der Beiträge.

Carolin Mogler

